# Supplementary material for: Are carbon emissions trading and green financial instruments synergistic? -Comprehensive quantitative research based on content analysis
Source: PLoS One. 2024 Mar 7;19(3):e0298601. doi: 10.1371/journal.pone.0298601 (PMC10919601; doi:10.1371/journal.pone.0298601)
Supplement: S1 Data — (ZIP) [file pone.0298601.s001.zip › Data/地级市Z_F工作报告.zip!/║╙─╧/┬σ╤⌠╩╨/└┤╫╘╣1⁄2╓┌║┼í╛┬φ┐╦╩2╛▌═°í┐_2010.docx]

各位代表：

现在，我代表市人民政府向大会作工作报告，请予审议，并请各位政协委员和列席人士提出意见。

一、克难攻坚，2009年我市经济社会实现了平稳较快发展

2009年是极不平凡的一年。受国际金融危机影响，年初我市经济增速大幅下滑，不少企业陷入困境，财政收支矛盾加大，加之长期积累的体制性、结构性矛盾集中凸显，经济社会发展面临着巨大压力和严峻挑战。一季度，全市生产总值增幅同比回落5.2个百分点；规模以上工业企业利润下降61%；财政一般预算收入下降14.6%。面对异常困难的局面，在省委、省政府和市委的正确领导下，在市人大、市政协的监督支持下，全市人民迎难而上，奋力拼搏，较好地完成了市十三届人大一次会议确定的各项目标任务，保持了经济社会平稳较快发展的良好态势。

——全市生产总值超2000亿元，经济总量实现新的跨越。初步核算，全年生产总值达到2075亿元，同比增长13%，分别高于全国、全省4.3个和2.3个百分点，用四年时间基本实现了经济总量翻一番。规模以上工业增加值830亿元，增长15.9%，高于全省1.3个百分点；实现利税277.3亿元、利润137.6亿元，分别增长24.6%和18.1%，高于全省15.4个和7.4个百分点；亏损企业亏损额下降69.9%，降幅居全省第二。财政一般预算收入120.3亿元，增长3.2%。全社会固定资产投资1447.3亿元、增长31.5%，其中工业投资798.3亿元、增长35%。社会消费品零售总额687.3亿元，增长19.1%。城镇居民人均可支配收入和农民人均纯收入实际分别增长9.7%和8.9%。金融机构年末存贷款余额分别达1719.7亿元和867亿元，比年初增加324亿元和203.6亿元，均创历史新高。

——项目带动战略加快实施，新企业、新园区、新基地建设取得明显进展。全年新开工千万元以上项目2649个、总投资1228.2亿元，同比增加851个、558.6亿元；其中亿元以上项目155个、总投资618.3亿元，同比增加79个、366.1亿元。在建千万元以上项目3049个、总投资1852.5亿元，同比增加918个、601.9亿元；其中亿元以上项目256个、总投资1126.2亿元，同比增加89个、398.8亿元。竣工千万元以上项目2498个、总投资660.6亿元，同比增加776个、167.8亿元；其中亿元以上项目87个、总投资123.3亿元，同比增加26个、6.5亿元。新谋划千万元以上项目1197个，总投资7827亿元。装备制造业方面，投资15亿元的南车集团洛阳机车公司城市轨道车辆组装服务基地、投资13.3亿元的一拖集团4万台重型柴油机和3.3万台大轮拖、投资11亿元的LYC轴承公司提升改造等项目开工建设，投资11.37亿元的新洛轴工程一期、投资8.8亿元的新河柴工程等项目进展顺利，投资4.2亿元的中信重工特大选矿装备、投资1.9亿元的725所高速铁路桥梁附件等项目竣工投产。有色金属产业方面，投资76亿元的新电集团40万吨铝板带箔、投资35亿元的龙鼎铝业60万吨铝板带箔、投资18亿元的香江万基氧化铝三期等项目开工建设，投资29.5亿元的伊电集团25万吨连铸连轧铝深加工、投资10亿元的钼都科技园、投资5.4亿元的725所5000吨钛板带等项目加紧建设，投资22亿元的中铝洛铜10万吨高精度电子铜板带、投资7.86亿元的洛钼集团4万吨钼冶炼一期等项目竣工投产。石化产业方面，投资26亿元的河南煤化集团20万吨乙二醇、投资4.6亿元的洛阳石化260万吨柴油加氢等项目开工建设，投资20.9亿元的洛阳石化45万吨PX、投资19亿元的吉利60万吨PTA等项目前期工作进展顺利。硅光电产业方面，投资20亿元的强生光电10×35兆瓦非晶薄膜太阳能电池、投资4.5亿元的5715厂光电研发中心等项目开工建设，投资13亿元的中硅公司2000吨多晶硅扩建、投资9.8亿元的613所光电研发中心等项目进展顺利，投资12.7亿元的昇扬硅业金属硅一期、投资8.5亿元的阿特斯2000吨长晶切片、投资7.9亿元的上海超日150兆瓦太阳能电池一期等项目竣工投产，投资25亿元的尚德公司1000兆瓦太阳能电池等项目正在积极谋划。能源电力产业方面，投资26亿元的新电集团2×30万千瓦热电联产机组等项目开工建设，投资52亿元的华阳孟津电厂2×60万千瓦机组等项目今年下半年可竣工投产，投资520亿元的中电投核电等项目正在积极谋划。新兴产业方面，投资35亿元的中航锂电产业基地、投资3.3亿元的河柴重工大功率煤层气发动机等项目加紧建设，投资5亿元的725所风电叶片产业基地一期、投资1.15亿元的普莱柯生物灭活疫苗等项目竣工投产。新园区不断壮大。中信重工、一拖集团、LYC轴承公司、河柴重工、中航光电等企业先后在产业集聚区建立了新园区。17家省级产业集聚区全年完成固定资产投资437亿元，建成标准化厂房190万平方米，新入驻企业258家；实现总收入2258亿元、利税198亿元。新基地迅速崛起。725所、613所、014中心、轴研科技、中色科技、黎明化工院等科研院所均在产业集聚区建立了研发和生产基地。

——农民生产生活质量持续改善，新农村建设扎实推进。农村经济稳步发展。认真落实惠农政策，全年发放各类生产性补贴和生活保障性补贴9.63亿元。战胜了几十年不遇的特大旱灾，全年粮食总产47亿斤，连续6年创历史新高。肉蛋奶总产73.2万吨、同比增长6%。烟叶、水果、蔬菜、核桃、中药材等特色种植业稳步增长。在第七届中国花卉博览会上，我市获金奖19项、占全省的63.3%，洛阳牡丹再夺桂冠。新发展涉农龙头企业、农民专业合作组织分别是全年目标的1.7倍和6.2倍。产业化收入占农民总收入的比重由48%提高到50%。集体林权制度改革全面推进。全年造林70.8万亩。转移农村劳动力146.9万人次，培训农民工23.4万人次，劳务创收75亿元。农业基础设施日益完善。孟西灌区总干渠全线贯通，引黄入洛工程顺利开工，小浪底南岸灌区工程稳步推进，前坪水库前期工作进展顺利。完成了24座病险水库除险加固任务。新增有效灌溉面积32万亩，改造中低产田5万亩。新农村建设扎实推进。新建乡级便民服务中心40个、农村社区服务中心213个、示范超市105个、标准化村卫生室1500个；建成农村公路1248公里，硬化通组通户道路3282公里。新建户用沼气5.5万座、沼气工程50座、沼气服务网点501个，在全省率先实现了沼气服务全覆盖。27.9万人的安全饮水问题得到解决。

——现代服务业快速发展，金融业和高端服务业实现新的突破。全市服务业增加值同比增长14.1%，分别高于全国、全省5.2个和3.2个百分点；对全市经济增长的贡献率达到32.3%，同比提高3.3个百分点。消费市场日趋活跃。投资25亿元的宝龙城市广场一期、投资7.6亿元的万达广场等7个大型商业项目建成开业。汽车、商品房等销售大幅增长，家电下乡产品销量占农户比例居全省第一。金融业发展势头强劲。中信、光大、兴业、民生等四大股份制银行，齐鲁证券、中原证券、安诚财险、民安财险等证券、保险机构相继入驻洛阳。洛阳银行成为全省率先跨区域经营的城市商业银行。全省首家农村商业银行在洛开业。累计建立政府融资平台53个，融资余额122.8亿元、较年初增加49.3亿元。累计成立担保公司86家，注册资本25.3亿元，提供融资担保60.7亿元。成立小额贷款公司11家，居全省首位。高端服务业发展迅速。平安保险数据中心、用友软件研发中心、太平洋门户网站开业后实现税收1300多万元，吸纳就业1000多人。中国移动河南客户服务中心（洛阳）、公路口岸物流中心信息服务平台开工建设。大一物流园区信息服务中心投入使用。

——重大节会活动圆满成功，文化旅游业持续增长。成功举办2009世界邮展暨第27届洛阳牡丹花会、河洛文化旅游节、大遗址保护高峰论坛、《秦汉－罗马文明展》等四大专题文明展。在全省率先组建了龙门、交通、航空三大旅游集团。龙门、小浪底西霞院、隋唐城遗址、汉魏故城四大文化旅游产业园区加快推进。全年接待游客5300万人次、其中入境游37万人次，景区门票收入5.7亿元，旅游总收入260亿元，同比分别增长20%、21%、14%和21%。

——重点领域改革步伐加快，发展活力不断增强。国企改革扎实推进。与中国黄金集团公司签订了战略合作协议，配合省政府完成了与国机集团、中航工业、中船重工、中国化工等央企战略合作协议的签订工作。全年引进增资扩股资金21.5亿元。一拖集团股权划转和增资扩股全面完成。洛玻集团减轻债务负担和搬迁改造工作取得阶段性成果。河柴集团、洛轴集团、春都集团、春都股份政策性破产和中钢洛耐、黄河水泥等依法破产工作取得新进展。一大批企业改革遗留问题逐步得到了妥善解决。文化体制改革等成效显著。成功组建洛阳日报报业集团。洛阳歌舞剧院、洛阳豫剧院转企改制工作已经完成。在全省率先整合组建了文化广电新闻出版局。政府机构改革正式启动。非公有制经济发展迅速。全年非公有制企业累计完成营业收入3495亿元，同比增长23.9%。非公有制经济对全市经济增长的贡献率由50.6%提高到52.1%。

——1000亿元大招商任务圆满完成，招商引资成效显著。成功举办2009世界邮展河南省情说明会暨第27届洛阳牡丹花会投资贸易洽谈会、河洛文化旅游节经贸洽谈会，组织参加第四届中博会等重大招商活动，全年签订招商引资合同500个，总投资1089.2亿元、增长26.6%。实际利用外资9.15亿美元、市外境内资金239.7亿元，实现了上年高基数上的持续增长。四季沐歌太阳能产业基地、万年硅业3000吨多晶硅等项目入驻洛阳。

——新区开发力度加大，城市承载能力明显提升。土地利用总体规划修编基本完成，第四期城市总体规划修编完成阶段性任务。全年完成城建投资174.1亿元，其中新区113.8亿元、洛河以北城区60.3亿元。新建道路22条，城市道路由707.1公里增加到746公里。新区开发力度加大。新区拓展区规划方案获省审批，新区空间战略规划、拓展区建设规划取得阶段性成果。新区核心区完成投资108亿元。厚载门街、新区污水总干管二期等项目即将竣工，新区医院、新区污水处理厂等项目加快推进。新区拓展区完成投资5.8亿元。伊河综合治理、龙顾路等23个千万元以上项目开工建设，撤村并城、职教园区、滨河商住园区等项目相继启动。新入驻服务业企业251家，占新区历年入驻企业总数的57.2%。洛河以北城区及县城改造步伐加快。瀛洲北路、汉宫路（道北二路）一期等11项工程竣工投用，华山北路、滨河北路（一期）等13项工程加紧建设。徐家营拆迁安置已经完成，西小屯、东涧沟整村开发加快推进。新开工商品房390万平方米、竣工355万平方米。洛河以北城区改造、城中村和城郊村改造、县城建设及改造分别完成投资25亿元、30亿元和47.7亿元，同比增长8.7%、100%和53.8%。气源工程加快实施。西气东输二线洛阳境干线工程、安阳至洛阳天然气管线开工建设，西气东输二线洛阳境支线前期工作进展顺利。

——交通基础设施项目加快推进，综合交通体系日趋完善。投资74亿元的洛栾高速、投资15亿元的洛阳至吉利快速通道、投资6.98亿元的洛阳至偃师快速通道、投资1.6亿元的洛阳至宜阳快速通道开工建设，投资30亿元的洛阳至洛宁高速、投资46亿元的尧山至栾川高速前期工作加快进行，连霍高速郑州至洛阳改扩建工程（洛阳段）进展顺利。郑西铁路龙门站基本建成。洛宜铁路支线改建工程开工建设。洛阳机场航站楼改造基本完成。城市和城际轨道交通前期工作进展顺利。瀛州桥、凌波桥、新西苑桥竣工通车，标志着洛河市区段规划的6座大桥全部建成，进一步拉大了城市框架，方便了群众出行。

——省定、市定实事顺利实施，民生问题有了新的改善。全力办好省定、市定实事，全年用于民生的支出达到了133.1亿元。就业工作不断加强。新增就业16.8万人、再就业7.4万人，分别是省定目标的1.8倍和2.1倍，在非常困难的情况下保持了就业形势的总体稳定。为1.1万人发放小额担保贷款5.1亿元，发放人数和发放总额均居全省首位。社会保障体系更加完善。全年发放城乡低保金4亿元，基本实现了动态管理下的应保尽保。彻底解决了27家政策性破产企业2.86万名退休人员的医保问题。在校大学生全部纳入城镇居民基本医疗保险范围。偃师市成为首批国家新型农村养老保险试点。129个村的整村推进扶贫任务超额完成。新建搬迁扶贫安置点49个、搬迁扶贫1万余人。保障性住房建设形成高潮。新开工廉租房、经济适用房、国有大企业职工住房改造30.8万、53.8万和30.5万平方米，建成10.2万、27.3万和15.7万平方米。在全省率先开展的农村困难群众危土窑洞、草房、土坯房改造任务提前完成。

——科技创新步伐加快，各项社会事业不断进步。自主创新能力不断提高。荣获国家科学技术进步奖6项，其中特等奖1项、一等奖1项、二等奖4项。新增各类研发机构36家，其中国家级工程技术研究中心1家、省级5家。中信重工矿山重型装备等4家实验室入选国家级重点实验室，实现了我省国家级重点实验室零的突破。我市成为首批国家创新型试点城市，高新区成为国家节能减排装备产业基地、首批国家新型工业化示范基地和国家硅材料及光伏高新技术产业化基地。文物保护展示成果显著。洛阳博物馆新馆、定鼎门遗址博物馆、汉魏故城北城墙展示等工程竣工投用，隋唐城宫城核心区拆迁整治完成，洛阳博物馆等7家博物馆免费开放。教育、卫生、文化等事业蓬勃发展。普通高招再创佳绩。新建和改造中小学11.2万平方米。新区洛一高加快推进，龙康中学、滨河中学、新区第二外国语学校等开工建设。甲型H1N1流感疫情得到了有效的控制，卫生应急工作跨入全国先进行列。新建文化信息资源共享工程县级中心5个、乡镇综合文化站23个、农家书屋230个。“十一五”广播电视村村通工程全部完成。成功举办市十一届运动会。创建国家森林城市工作通过国家验收。人口自然增长率控制在4.87‰。我市援建四川江油中坝镇恢复重建项目全部竣工。民族宗教、外事侨务、统计、审计、人防、气象、口岸、地震、地方志、残疾人事业等工作都取得了新成绩。

——精神文明和民主法制建设不断加强，社会大局保持稳定。创建全国文明城市取得了阶段性成效。驻洛部队和民兵预备役为洛阳发展做出了突出贡献，我市荣获省双拥模范城六连冠。自觉接受人大及其常委会和政协的监督，人大代表议案、建议和政协委员提案办结率100%、满意率98%以上。提请市人大及其常委会审议地方性法规3件，发布政府规章3件。严查违法乱纪案件，反腐败斗争取得新的成效。建立矛盾纠纷立案交办等制度，有效化解了一批影响社会稳定的突出问题。严格落实安全生产责任制，实现了事故起数、死亡人数、受伤人数、直接经济损失“四下降”。加强基层基础工作，完善社会治安防控体系，社会大局保持稳定。

经过全市人民的共同努力，我市先后荣膺全国节能减排二十佳城市、最具投资价值的中国新能源产业城市、全国可再生能源建筑应用示范城市、全省平安建设先进市、央视网首届中国精彩城市等称号，再获全国科技进步先进市殊荣，被国土资源部授予“双保行动”先进单位，城市知名度和影响力大幅提升。

各位代表！过去的一年，面对前所未有的困难和挑战，655万洛阳人民始终保持昂扬向上、奋发有为的精神状态，齐心协力，克难攻坚，取得了战危机、保增长的阶段性胜利。我们坚持把保持经济平稳较快发展作为政府工作的重中之重，先后出台了60条和46条应对危机、加快发展的意见和26个配套政策，着力打好六大攻坚战、落实好十大重点任务、实施好十大行动计划、推进好十大提速工程、实施好十大保障措施。我们坚持把项目建设作为保增长、调结构的有效载体，积极争取中央、省政策资金28.5亿元，将原定三年财政投资项目集中到一年建设，一批大项目、好项目顺利实施，我市在全省重点项目观摩活动中名列前茅。我们坚持把服务企业作为保增长、促发展的着力点，深入开展企业服务年活动，帮助企业解决突出问题582个，特别是在财政形势异常困难的情况下，积极为企业办理减免退税46亿元、占全省的19%，虽然影响了我市财政收入增速，但对企业的发展起到了很大的帮助作用。我们坚持把新区开发与洛河以北城区改造相结合、县城改造与小城镇建设相结合、新农村建设与城乡一体化相结合，进一步拓展了发展空间。我们坚持把改善民生作为一切工作的出发点和落脚点，越是在困难的形势下越是高度重视民生问题，同时下大力解决好安全生产、信访稳定等深层次矛盾，以发展促和谐、以和谐求发展的氛围进一步浓厚。

这些成绩的取得，是省委、省政府和市委审时度势、正确决策的结果，是市人大、市政协鼎力支持、密切协作的结果，也是全市人民同心同德、攻坚克难的结果。在此，我代表市人民政府，向辛勤工作在各条战线的全市人民，向人大代表、政协委员，向各民主党派、人民团体，向离退休老领导、老同志，向驻洛部队、武警官兵和公安干警，向所有关心支持洛阳发展的社会各界和海内外朋友，表示衷心的感谢并致以崇高的敬意！

肯定成绩的同时，还要清醒地看到，我市经济社会发展中仍存在不少问题，我们面临着保增长与调结构、促转型、增效益、保稳定的多重压力：一是产业结构不尽合理。工业资源型、基础性产业比重大，产品链条短，增值能力弱；多数企业自主创新能力不强，竞争力较弱，高技术产业增长乏力。二是经济增长的质量有待进一步提高。受国际金融危机、退税政策等因素影响，财政收入增速较低，增收能力弱与刚性支出大的矛盾依然突出；外需持续低迷。三是统筹经济社会协调发展的难度加大。随着经济社会发展步入转型期，各种利益诉求增多，劳动就业、社会保障、教育、卫生、住房、安全生产等关系人民群众切身利益的问题尚未得到很好的解决。四是政府工作水平有待进一步提高。个别同志不能适应快速发展的形势，观念转变缓慢，工作方式粗放；个别部门服务意识不强、工作效率不高，发展环境需要进一步优化。对此，我们一定要采取有效措施，认真加以解决。

二、理清思路，科学制定2010年我市经济社会发展目标

2010年是决战“十一五”、谋划“十二五”的关键之年。当前，我们面临着加快发展的诸多有利条件和重要机遇。国际金融市场和实体经济趋稳向好的轨迹越来越明显，世界经济正在逐步走出危机。我国经济初步实现了“V”型复苏，经济企稳回升的态势已基本形成。今年中央继续保持宏观经济政策的连续性和稳定性，在工业、农业、服务业、基础设施、社会事业等方面出台了一系列的支持政策，国家启动中部振兴规划，为我们加快发展创造了有利的政策环境。同时也要看到，我市经济发展仍然面临着很多困难和挑战。国际金融危机影响依然存在，世界经济复苏基础仍不稳固，外需低迷的状况难以在短期内改变，区域竞争压力与资源瓶颈约束等相互叠加，结构性矛盾仍很突出，经济发展内在动力仍然不足，将继续对我市产生重要影响。我们一定要准确把握，趋利避害，扬长避短，科学应对，真正把各种机遇转化为加快发展的新优势，努力把政府各项工作做得更好，决不辜负全市人民的殷切期望！

2010年政府工作的总体思路是：全面贯彻党的十七届四中全会和中央、省委、市委经济工作会议精神，深入贯彻落实科学发展观，坚持“四个重在”，以转变经济发展方式和调整经济结构为主线，以保增长、保民生、保稳定、保态势为着力点，大力实施工业强市、旅游强市、科教强市和项目引资双带动战略，全面加快项目建设、城镇建设和民生建设，进一步深化改革、扩大开放，不断提高发展质量和效益，努力实现经济社会平稳较快发展。

全市经济社会发展的主要预期目标是：地区生产总值增长11%；财政一般预算收入增长10%；全社会固定资产投资增长20%以上；社会消费品零售总额增长16%以上；外贸出口增长11%，实际利用外资增长14%；城镇居民人均可支配收入实际增长7.5%，农民人均纯收入实际增长6.5%；单位生产总值能耗下降4.5%；新增城镇就业10万人，城镇登记失业率控制在4.5%之内；人口自然增长率控制在6.5‰之内。

实现上述目标，必须把握好以下六个方面。一是在保持经济平稳较快发展的同时，更加注重调整经济结构和转变发展方式，努力实现可持续发展；二是在实现规模扩张的同时，更加注重提高质量和效益，努力实现优质发展；三是在扩大投资和出口的同时，更加注重扩大消费、提升服务业发展水平，努力实现均衡发展；四是在加快经济建设的同时，更加注重节能减排和生态建设，努力实现绿色发展；五是在提升城市形象品位的同时，更加注重加快城乡一体化进程，努力实现协调发展；六是在发展经济的同时，更加注重改善民生和保持社会稳定，努力实现和谐发展。

三、扎实工作，确保圆满完成“十一五”各项目标任务

坚持把保增长作为政府工作的首要任务，把调结构、促转型作为经济工作的主线，全面加快“一个载体、三个体系”建设，下大力改善民生，确保圆满完成“十一五”各项目标任务。

（一）加快项目建设，推动产业转型升级。力争全年完成固定资产投资2000亿元以上，计划新开工千万元以上项目超过3500个、总投资1800亿元以上；竣工千万元以上项目超过3000个、总投资1100亿元以上。其中，计划新开工千万元以上工业结构调整项目331个、总投资869亿元；竣工千万元以上工业结构调整项目333个、总投资802亿元。推动工业提升质量。一是做大做强优势产业。以调高、调优、调强为目标，壮大装备制造业，提高有色产业加工度，延伸石化产业链条，提升硅光电产业整体水平，培育一批核心竞争力强的大企业、大集团。筛选一批技术含量高、单位产出率高、创新能力强、带动能力强、能源消耗低、环境污染小的重大项目，争取进入省“转型升级双千工程”，确保年内全部开工。重点抓好中航锂电产业基地、河南煤化集团乙二醇、南车集团洛阳机车公司城市轨道车辆组装服务基地、强生光电非晶薄膜太阳能电池、一拖工业园、新洛轴工程、龙鼎铝业60万吨铝板带箔等127个重大项目，尽快竣工投产。积极谋划实施1万亿元重大项目，重点抓好核电、大鱼沟抽水蓄能电站、洛阳石化新增1000万吨炼油等项目前期工作。二是培育发展新兴产业。加快国家兽用药品工程技术研究中心建设以及中药现代化培育，依托中硅公司、尚德公司、阿特斯公司多晶硅及切片、太阳能电池组件等项目发展太阳能光伏产业，依托中信重工纯低温余热发电设备、725所风电叶片等项目发展清洁能源发电设备产业，努力构建低碳排放、就业充分、综合效益好的现代产业体系。

推动现代服务业扩充总量。制定现代服务业和物流业发展规划，着力推进呼叫产业园区、龙门文化旅游产业园区等6个省级服务业特色园区基础设施和公共服务平台建设。重点抓好40个投资3000万元以上的服务业项目，全年完成投资50亿元。加快无线洛阳建设，基本实现 3G 网络全覆盖。启动电子政务网络整合、企业基础信息共享、文化信息资源共享和公共卫生信息系统等工程。加快拖拉机质量检测鉴定中心、高新区专利成果转化服务平台、轴承产业发展服务平台等项目建设。鼓励支持工业企业分离设计、物流、购销、售后服务等环节，发展生产性服务业。积极发展互联网、软件、电子商务企业。抓好中国移动河南客户服务中心（洛阳）、轴承物流园区、洛阳国际（口岸）物流园区等项目建设。

推动文化旅游业再上水平。完善旅游业发展规划，加快转型升级，打造最佳旅游目的地城市。突出抓好隋唐洛阳城、汉魏故城等重点旅游功能区建设，推进伏牛山滑雪度假区二期、玄奘故里旅游区、伏牛山白河游客服务中心等项目建设，打造龙门世界级旅游精品景区。抓紧建设郑西铁路龙门站游客服务中心，开通观光巴士和景区直通车，实现旅客零换乘。加强空港建设，增加旅游航班、航线。完善旅游信息平台、城市标识系统、旅游集散中心功能。办好牡丹花会和河洛文化旅游节。发展壮大龙门、交通、航空等旅游集团。加强旅游宣传推介，拓展旅游线路，丰富旅游产品。抓好小浪底西霞院、龙门文化旅游产业园区，平乐牡丹画专业村等十大文化产业示范村，《洛神》等八大重点文化产业项目。大力发展演出业、娱乐业，开发文化新业态、新产品，打造优秀动漫创意产品。

推动产业集约、集聚发展。以新材料、节能环保等国家级产业基地和洛阳动力谷、17个省级产业集聚区为依托，加快新企业、新园区、新基地建设，超前谋划更多后续项目，争取更多产业集聚区列为省新型工业化产业示范基地。严格落实国家耕地、基本农田保护和节约集约利用政策。实行产业集聚区建设用地指标重点保障和环境容量指标优先配置，健全投融资平台、土地开发整理中心，加快发展配套服务业。实施产业集聚区投资倍增计划，全年完成投资600亿元以上，其中基础设施投资80亿元、产业投资520亿元。

（二）注重质量效益，保持经济平稳较快增长。加强经济运行协调。密切关注宏观经济走向，完善运行监测体系，及时发现并马上解决苗头性问题。完善统筹协调机制，提高煤电油气运和资金等生产要素保障能力。大力实施企业服务行动计划，市、县两级分别抓好100家重点企业，推动服务企业工作常态化。实施名牌带动战略，加强营销体系建设，组织产销对接会、供需见面会，帮助企业开拓市场。

培育新的消费热点。落实好义务教育学校绩效工资、困难群众补贴救助等政策，健全企业工资集体协商制度和工资支付保障机制，不断提高居民消费能力。扩大住房、汽车等大宗消费，推动家电等耐用消费品升级换代，大力发展社区物业、家政、社会养老等服务性消费，积极开发旅游、文化、体育健身和网络等热点消费。开发一批重点商圈、特色商业街，开工建设铜锣湾广场、摩天中城商业广场等项目，加快推进正大广场、郑西铁路龙门站商圈、新区万达商业项目等。落实家电、汽车、摩托车下乡，减征汽车购置税，汽车以旧换新等消费政策，销售家电下乡产品22万台以上，汽车及摩托车下乡产品4万台。加快万村千乡市场工程、双百市场工程建设，新建、改造农家店500个。规范房地产建设行为，积极打造洛阳房地产品牌，促进房地产业健康发展。

增强财税金融保障能力。加强收入分析和税源监管，积极培育新的税收增长点，严格执行非税收入“收支两条线”，确保应收尽收。优化财政资源配置，强化预算管理，严格控制一般性支出，集中财力保民生、保运转、保重点。落实增值税转型等税收政策，大力支持结构调整、节能减排、自主创新和现代产业体系建设。组织好银企洽谈会、项目推介会，引导金融机构创新金融产品，扩大信贷投放。加快发展中小企业贷款担保机构和小额贷款公司，力争中小企业贷款增速不低于全部贷款的平均增速。支持洛阳银行增资扩股，争取组建一批村镇银行；吸引金融、保险、证券企业来洛开设分支机构。支持新电集团、北方玻璃、隆华制冷、通达电缆等企业尽快上市。

（三）坚持改革创新，转变发展方式。实施重点改革行动计划。一是深化国有企业改革。加快推进国企战略重组，争取与2家以上央企签订重大战略合作协议。抓好洛轴集团、春都集团、春都股份等企业的政策性破产工作。力争年底前基本完成物产集团、明珠热电、中钢洛耐等企业的依法破产审理程序。加快实施洛玻集团振兴工程和白马集团异地搬迁改造工程。妥善解决中小企业改制遗留问题。强化国有资产监督管理，确保国有资产收益最大化。二是积极推进其它领域改革。深化行政管理体制改革，加快市、县政府机构改革，稳妥推进事业单位分类改革和机关事业单位收入分配制度改革。积极推进文化体制改革，全面启动国有文艺院团转企改制。实施医药卫生体制改革，启动医疗救助与新农合、城镇职工医保、居民医保同步结算工作。搞好公立医院改革试点、医疗保险市级统筹试点和异地就医结算试点。深化财税金融体制改革，推进资源性价格改革和环保收费改革。深化集体林权制度改革和县级供销社改革。引导农村土地有序流转，提升土地规模经营水平。

着力提升创新能力。一是突破一批关键技术。实施科技支撑产业升级工程，重点支持装备制造、新材料、新能源、信息产业、生物产业等领域的关键技术研发，实施好特种精密轴承、新能源电动汽车、盾构机、优质高效耐火材料等重大科技专项。二是推进一批创新成果产业化项目。抓紧编制高性能结构材料、纳米材料和器件技术等发展规划，实施好高效晶体硅太阳能电池产业化、兆瓦级风力发电机组轴承、锂离子电池、超级电容等重大科技项目。力争全年高技术产业增加值增长13%以上。三是打造一批科技领军企业。全年培育高新技术企业20家，争取更多的企业成为国家、省创新型企业。确保高新技术企业总产值突破500亿元，同比增长20%。实施知识产权推进战略，全年专利申请量增长30%以上，努力实现由产品制造向品牌创造转化。四是构建完善的创新体系。探索建立风险投资平台，多渠道增加研发投入，新增研发机构20家。完善科技创新技术服务平台，加快引进天使基金、创投基金，推动创业孵化器和创业中心建设，建立产业技术创新联盟。完善人才激励机制，培养、引进高层次科技人才。五是加快国家创新型试点城市建设。重点抓好国家新型工业化产业示范基地、国家知识产权工作示范城市、国家新材料高技术产业基地、国家级重点实验室和工程技术研究中心建设，力争新建1－2个国家级重点实验室和工程技术研究中心，在优势领域新建1－2个博士后工作站。实施好国家“金太阳示范工程”，加快省级硅材料光伏特色产业基地建设。

加强节能减排和生态建设。一是完善工作机制。加大节能减排投入，健全节能减排工作责任制，确保全年单位GDP能耗下降4.5%、化学需氧量和二氧化硫排放量分别控制在4.01万吨和21.7万吨以内。加强建设项目审批管理，严控高污染、高耗能项目，加速淘汰落后产能。二是抓好重点工程。实施电力、钢铁、有色、建材、石化、煤炭等九大重点行业节能攻坚计划，推进燃煤锅炉改造、热电联产、建筑节能、绿色照明、政府机构节能等十大重点节能工程，加快有色金属、建材、石油加工等企业减排项目建设。依托国家可再生能源建筑应用示范城市建设，实施太阳能利用示范小区等39项节能建筑示范工程，争取城市公交、出租、电动汽车示范运营国家试点。大力推进节水技术改造和节水工程。三是加大环保力度。加强重点流域、重点区域、重点行业、重点企业污染综合整治和水源地保护。加快二、三期污水处理厂建设，抓好非电行业脱硫治理、重点涉水行业深度治理，全面建成环境自动监控系统。加强农村环境保护，控制农村面源污染。四是发展循环经济。抓好大唐洛热10兆瓦太阳能发电项目前期工作，加快工业、农产品加工废弃物资源化等循环经济工程建设，培育一批产业集聚区、企业等循环经济试点。五是加强生态环境建设。实施绿色碳汇工程，抓好林业生态市建设，加快城郊绿化、荒山绿化、通道绿化和村庄绿化。抓好洛河市区段湿地公园建设，实施涧河市区段治理，构建新型滨水生态景观带。

（四）进一步加大开放力度，切实增强发展活力。注重招商引资实效。以珠三角、闽东南、长三角、环渤海湾、港澳台、东南亚等地区为重点，开展定向招商活动，引导电子、服装、玩具等产业向我市转移。扩大与国内外优强企业的战略合作，下大力引进一批成长性好、关联度高、示范带动力强的产业链延伸项目和科技型、龙头型企业。加快发展服务外包企业，引进服务外包项目入驻产业园区。建立外来企业投资重大项目联系跟踪机制，鼓励外来投资企业增资扩产，提高招商合同履约率和外资到位率。重点抓好113个亿元以上、总投资1004.7亿元的在谈项目，183个亿元以上、总投资986.3亿元的签约项目，282个亿元以上、总投资1336.3亿元的在建项目。

保持外贸外经稳定增长。用足用好外贸发展专项资金，培育一批具有自主品牌和出口规模的大型出口企业，争取4家企业进出口超亿美元。实施科技兴贸和机电产品出口推进计划，支持企业开发新产品，重点培育2个出口超亿美元、20个出口超千万美元的拳头产品。深度开发欧美日等传统市场，大力拓展非洲、中东、俄罗斯等新兴市场。提高对外工程承包和劳务合作水平，带动设备和技术出口。

大力发展非公有制经济。认真落实支持非公有制经济发展的各项政策措施，积极争取中小企业发展专项资金，加快中小企业信息服务网络和创业基地建设，鼓励各类主体兴办个体、私营企业，支持非公有制企业加快项目建设，重点扶持一批主业突出、高成长性、竞争力强的企业。新上投资500万元以上非公有制项目450个，其中超千万元项目260个、超亿元项目50个。规模以上非公有制企业达到1350家，其中超10亿元企业10家、超5亿元企业30家、超亿元企业130家。

（五）加快新农村建设，切实做好“三农”工作。认真落实惠农政策。按照中央1号文件要求，加大“三农”投入，及时发放粮食直补、农资综合补贴、良种补贴、农机具购置补贴，认真执行粮食最低收购价等政策，切实把中央各项支农惠农政策落到实处。大力发展现代农业。坚持以市场为导向、农业增效为中心、农民增收为目标，推进特色优势农业产业化，实现传统农业向现代农业的转变。实施粮食稳产保收行动计划，抓好粮食核心区主产县建设，确保全年粮食总产稳定在40亿斤以上。抓好近郊和周边蔬菜、食用菌、牡丹等基地建设，稳定烟叶种植面积，新建核桃基地8万亩。加快优质畜产品生产加工基地建设和规模养殖场标准化改造升级，新增牛奶加工能力3万吨、肉制品加工能力5万吨，肉蛋奶总产达到80万吨以上。新增省级龙头企业20家以上、争取国家级1－2家，发展各类农民专业合作社50家。完成农村劳动力转移培训20万人次，转移输出135万人次，创收70亿元。加强农业基础设施建设。争取前坪水库尽快立项，引黄入洛工程上半年全线开工；抓紧完成15座水库除险加固工程，加快实施孟津黄河渠等中型灌区及水利设施修复配套工程。新增和改善灌溉面积8万亩，改造中低产田5万亩，建设高标准粮田5.2万亩。扎实推进新农村建设。建好100个市级示范村，搞好200个重点整治村基础设施建设。开工建设20个市级新型农村社区，新增农村社区服务中心100个。修建农村公路120公里，硬化通组通户道路2000公里。发展农村户用沼气3万户，新建各类沼气工程40座、沼气服务网点300个。解决安全饮水15万人，新增农村自来水受益人口20万人。完成100个村的整村推进和1万人的搬迁扶贫任务。新建、改扩建农村敬老院25所。为2979个行政村放映电影3.57万场，免费文化演出450场；为195个村庄各建一个篮球场、两个乒乓球台。

（六）加快城镇化进程，提升辐射带动能力。搞好城乡规划。加快第四期城市总体规划和土地利用总体规划修编报批工作，完善交通、人防、抗震防灾、防洪排涝等专项规划，推进优秀近现代建筑保护利用规划，做好伊河、洛河两岸城市单体建筑规划设计，塑造鲜明城市特色。严格规划执法，确保规划执行的严肃性。

完善城市功能。探索建立多元化的城市基础设施经营管理体制，重点在综合交通体系、能源基地、安居工程、社会事业等领域实施400个重大项目，力争新开工项目190个、竣工187个。完成洛阳机场改扩建工程。开工建设新郑机场－登封－洛阳城际铁路。做好焦作－济源－洛阳城际轨道、市区轨道交通项目前期工作。抓好“四站、四桥、三通道”工程（公路运输枢纽客运南站，汽车长途客运东站，新区公交车站，郑西铁路龙门站南站房；华山路跨涧河桥、跨陇海铁路立交桥，洛偃快速通道跨伊河大桥，老洛阳桥改扩建；洛阳至洛宁高速，洛阳至栾川高速，洛阳至吉利快速通道），尽快开工连霍高速洛阳至三门峡改扩建工程（洛阳段），争取上半年开工洛阳至洛宁高速，洛栾高速嵩县至栾川段前期工作要取得实质性进展、力争年底前启动。完成华山路一期等扩宽改造和丽春东路、芳林南路、唐宫西路打通等50项市政基础设施建设。启动中心城区过街天桥和快速通道建设。抓好西气东输二线主干线洛阳段和境内支线，安阳－洛阳天然气管道洛阳段及17项电网项目。

加快新区建设。核心区方面，要突出抓好开元大道东段片区、洛龙科技园区、郑西铁路龙门站周边地区、关林商贸市场片区等四个片区和九个“十大工程”，确保完成投资150亿元。开工建设高铁大道、广阜路等，完善龙门站周边道路基础设施。加快新区图书馆等公用设施建设，确保新区医院花会前开业、集中供热管网“十一”前建成。加强市场规划建设，搞好配套服务。拓展区方面，抓好新区空间发展战略分区规划、局部控制性详规和市政、公共服务设施等专项规划编制。加快伊河综合治理、伊滨公园、伊洛大道、希望路、洛偃快速通道、撤村并城样板小区、职教园区、产业集聚区等项目建设，形成明显的形象进度，确保完成投资50亿元。

彰显城市文化特色。在城市规划、建设中更多地融入历史文化元素，更加注重城市建筑的单体设计，充分体现洛阳厚重的文化特色和地域特色。做好历史文化街区、工业文化遗产的保护与展示。完善大遗址保护规划，抓好隋唐洛阳城、汉魏故城等大遗址保护展示工程，基本完成明堂、天堂遗址保护展示工程。筹建二里头、偃师商城遗址公园。加快丝绸之路及大运河申报世界文化遗产工作。

提升精细化管理水平。积极开展“城市管理年”活动，完善城市管理长效机制，建立数字化城市综合管理系统，实施精细化、网格化管理，实现管理重心下移。提升物业管理水平，城市区物业管理覆盖率达到100%。加大公共交通投入，新购公交车300辆，新开通公交线路5条以上，优化调整公交线路5条，加密公交线路15条。实施城市交通综合整治工程，提高城市畅通能力。

加快县城新区和小城镇建设。科学编制县城新区规划，确保年内县城新区建设全部启动。县城改造、建设计划投资30亿元，完成拆迁130万平方米，开工建设270万平方米。积极开展特色中心镇试点建设，完善路、水、热、垃圾处理等公共服务设施，发展工业、商贸、文化、餐饮、娱乐等产业，吸纳农村人口向小城镇转移。抓好偃师、嵩县、栾川城乡一体化试点，加速城乡一体化进程，力争全市城镇化率达到46%。认真落实鼓励农民进城定居优惠政策，有步骤地解决农民工就业和生活问题。

（七）加强民生工程建设，全面构建和谐洛阳。办好实事惠民生。坚持把民生工作摆在更加突出的位置，大幅增加民生投入，认真办好省定、市定实事。对城市居民，要充分运用住房公积金支持保障性住房建设试点城市政策，新开工廉租房、经济适用房21万平方米和45万平方米，竣工16万平方米和30万平方米；城市棚户区、工矿棚户区（国有大企业职工住房）改造新开工50万平方米、竣工15万平方米。洛河以北城区及城中村改造完成拆迁130万平方米，开工300万平方米，竣工200万平方米。实施24条城市区背街小巷的路面改造和路灯照明改造工程。市区东部热源厂2011年春节前向用户供暖。逐步解决居民水电一户一表改造问题。新增放心早餐营业网点400个。新建信报箱40万个，覆盖率达到90%。对农村居民，要加快新型社区、农村公路和通组通户道路、安全饮水、沼气工程、敬老院、搬迁扶贫等工程建设，不断改善农村生产生活条件。

扩大就业惠民生。实施就业促进行动计划，城镇新增就业10万人、再就业4万人，其中就业困难人员实现就业1.5万人。新开发公益性岗位1200个，公益岗位在岗人数达到1万人以上。完善支持自主创业和自谋职业的各项政策，全年为创业人员发放小额担保贷款6亿元以上。开展大学生就业帮扶安置专项行动，强化对“4050”人员、失业人员和农民工的就业服务。

提高社会保障水平惠民生。扩大企业养老、失业、工伤和生育保险覆盖面，基本解决关闭破产国有企业退休人员医疗保障问题，城镇居民基本医疗保险参保人数达到90万人，争取更多县区进入新型农村社会养老保险改革试点。提高新农合保障水平，参合农民人均筹资达到150元，各级财政补助不低于120元。做好城乡低保工作，实现动态管理下的应保尽保。完善被征地农民补偿和保障机制。认真落实困难群众救助、五保户供养等补贴政策，提高优抚对象抚恤、补助标准。探索制订农民工享受城市居民社会保障待遇的政策。

发展社会事业惠民生。加强薄弱学校建设，整合教育资源，促进教育均衡发展。实施农村义务教育教师特设岗位计划，提高义务教育水平。实施职业教育攻坚计划，加快示范性职业院校和职业教育实训基地建设，基本完成中等职业学校布局调整。积极支持高等教育发展。实施校舍安全工程，确保完成三年改造任务的90%。加强新区学校、农村学校建设，尽快开工建设龙泰中学、龙瑞中学、龙祥中学和吉利初中、史家屯中学，确保新区洛一高、龙康中学、滨河中学今年秋季投用。办好河南省第十一届运动会。实施优势医院倍增发展计划，完善农村三级卫生服务网络和城市社区卫生服务体系，抓好重大传染病防控与救治工作。大力发展老龄事业，提高养老服务水平。稳定低生育水平，统筹解决人口问题。

建设平安洛阳惠民生。实施安全发展行动计划，建立安全生产投入保障机制，落实安全生产责任制，加强重大安全隐患排查和整治，严格重点领域、重点行业安全监管，推进小煤矿兼并重组和关闭等工作，杜绝重特大安全事故发生。健全信访工作长效机制，落实矛盾纠纷立案交办等制度，努力把不稳定因素化解在基层、消除在萌芽状态。加强社会治安综合治理，实施技防全覆盖工程，加大公共场所巡逻管控力度，强化流动人口服务管理，有效遏制各类案件发生。深入开展打黑除恶专项行动，保持对违法犯罪活动的高压态势。加强网吧监管，预防和减少青少年违法犯罪。对困难群体提供司法救助和法律援助。

创建文明城市惠民生。积极创建全国文明城市，加大硬件投入，加快科技馆、群艺馆、青少年活动中心建设，提升档案馆建设管理水平，力争文明城市测评取得新突破。深入开展“三优三创”竞赛活动，提高市民文明素质，营造健康向上的人文环境。支持驻洛部队和民兵预备役建设，落实优抚安置政策，搞好双拥共建。依法保护妇女儿童、残疾人和未成年人合法权益。

（八）编制好“十二五”规划，努力在新一轮发展中抢占先机。密切关注政策走向，及时了解国家、省“十二五”规划编制信息，加强与上级规划的衔接，着力提高规划的前瞻性、指导性和可操作性。紧紧围绕经济社会发展的主要矛盾和突出问题，加强战略性重大专题研究，组织开展专题调研、专家访谈、市民建言献策等活动，确保规划科学有效、符合民意；搞好总体规划与专项规划、新规划与已有规划的统筹衔接，确保规划的连续性、协调性。积极谋划一批前景好、潜力大、带动力强、科技含量高的好项目，争取更多项目进入国家、省规划盘子，争取更多政策、资金支持。

（九）加强政府自身建设，打造人民满意政府。强化服务意识。牢固树立执政为民的理念，扎实开展“两转两提”活动。要把为基层、为企业、为群众提供良好公共服务作为政府工作的首要任务，一切工作都要以群众为中心，以群众需要为第一选择、以群众利益为第一追求、以群众满意为第一标准，做到工作责任到位、政策落实到位、措施保障到位。要通过我们的实践，真正体现政府是人民的政府，政府是真正为老百姓服务的政府，每个公务员都是人民群众的服务员。

提高服务能力。加强学习型政府建设，探索建立多层次、多渠道、多形式、重实效的公务员培训长效机制。重点加强宏观政策、市场经济、依法行政、城市建设和管理等方面的培训，进一步更新观念，开阔视野。坚持双休日正常办理请办事项等制度，确保基层请办事项有报必理、有理必果、有果必复。从今年起，在全市各级行政机关推行首问负责制，凡与基层、企业、群众打交道多的部门都要设立首问负责科，为基层、企业、群众提供一站式、全过程服务。

提高行政效能。创新政府管理和服务方式，进一步下放管理权限，推进行政审批权相对集中改革，搞好项目联审联批，精简审批项目，提高审批效率。认真落实请办事项限时办结制度，加强电子政务建设，不断提高行政效能。完善绩效考核评价体系，强化行政问责，坚决纠正行政不作为和乱作为。

坚持依法行政。严格执行人大及其常委会的决议和决定，自觉接受市人大及其常委会的法律监督、工作监督和政协的民主监督，认真办理人大代表议案、建议和政协委员提案。广泛听取人大代表、政协委员和民主党派、工商联、无党派人士意见，充分发挥人民团体的桥梁纽带作用。严格按照法定权限和程序行使权力、履行职责。完善协同执法机制，提高执法水平。加强政府信用建设，提高政府公信力。

坚持廉洁从政。加强惩治和预防腐败体系建设，认真落实廉政建设责任制，严格执行部门预算、政府采购等制度，从源头上预防腐败。严格控制楼堂馆所建设和公费出国出境，坚决制止铺张浪费。严肃查处违法违纪案件，保持惩治腐败的高压态势。严格要求、严格教育、严格管理、严格监督干部，做到堂堂正正做人、清清白白做官、干干净净做事。

各位代表，新的一年孕育新的希望，新的一年肩负新的使命。让我们紧密团结在以胡锦涛同志为总书记的党中央周围，坚定信心，勇往直前，努力创造无愧于时代、无愧于历史、无愧于人民的新业绩，建设一个更加富强、更加美丽、更加和谐的新洛阳！
